# Transanal circumferential pouch advancement for treatment of pouch vaginal fistulae

**DOI:** 10.1007/s10151-024-02983-7

**Published:** 2024-08-14

**Authors:** M. Obi, M. Klingler, I. Sapci, O. Lavryk, J. Lipman, S. R. Steele, T. Hull, S. D. Holubar

**Affiliations:** 1 Department of General Surgery, Digestive Disease and Surgery Institute, Cleveland Clinic, Cleveland, OH USA; 2 Department of Colon and Rectal Surgery, Digestive Disease and Surgery Institute, Cleveland Clinic Main Campus, 9500, Cleveland, OH 44122 USA

**Keywords:** Ileoanal pouch, Ulcerative colitis, Perianal Crohn’s disease, Fistula-in-ano, Rectovaginal fistula, Pouch salvage

## Abstract

**Background:**

Ileal pouch anal anastomosis (IPAA) circumferential pouch advancement (CPA) involves full-thickness transanal 180–360° dissection of the distal pouch, allowing the advancement of healthy bowel to cover the internal opening of a vaginal fistula. We aimed to describe the long-term outcomes of this rare procedure.

**Methods:**

Patients with IPAA who underwent transanal pouch advancement for any indication between 2009 and 2021 were included. Demographics, operative details, and outcomes were reviewed. An early fistula was defined as occurring within 1 year of IPAA construction. Clinical success was defined as resolution of symptoms necessitating CPA, pouch retention, and no stoma at the time of follow-up. Figures represent the median (interquartile range) or frequency (%).

**Results:**

Over a 12-year period, nine patients were identified; the median age at CPA was 41 (36–44) years. Four patients developed early fistula after index IPAA, and five developed late fistulae. The median number of fistula repair procedures prior to CPA was 2 (1–2). All patients were diagnosed with ulcerative colitis at the time of IPAA and all late patients were re-diagnosed with Crohn’s disease. Four (44.4%) patients had ileostomies present at the time of surgery, three (33.3%) had one constructed during surgery, and two (22.2%) never had a stoma. The median follow-up time was 11 (6–24) months. Clinical success was achieved in four of the nine (44.4%) patients at the time of the last follow-up.

**Conclusions:**

Transanal circumferential pouch advancement was an effective treatment for refractory pouch vaginal fistulas and may be offered to patients who have had previous attempts at repair.

## Introduction

**Fig. 1:**
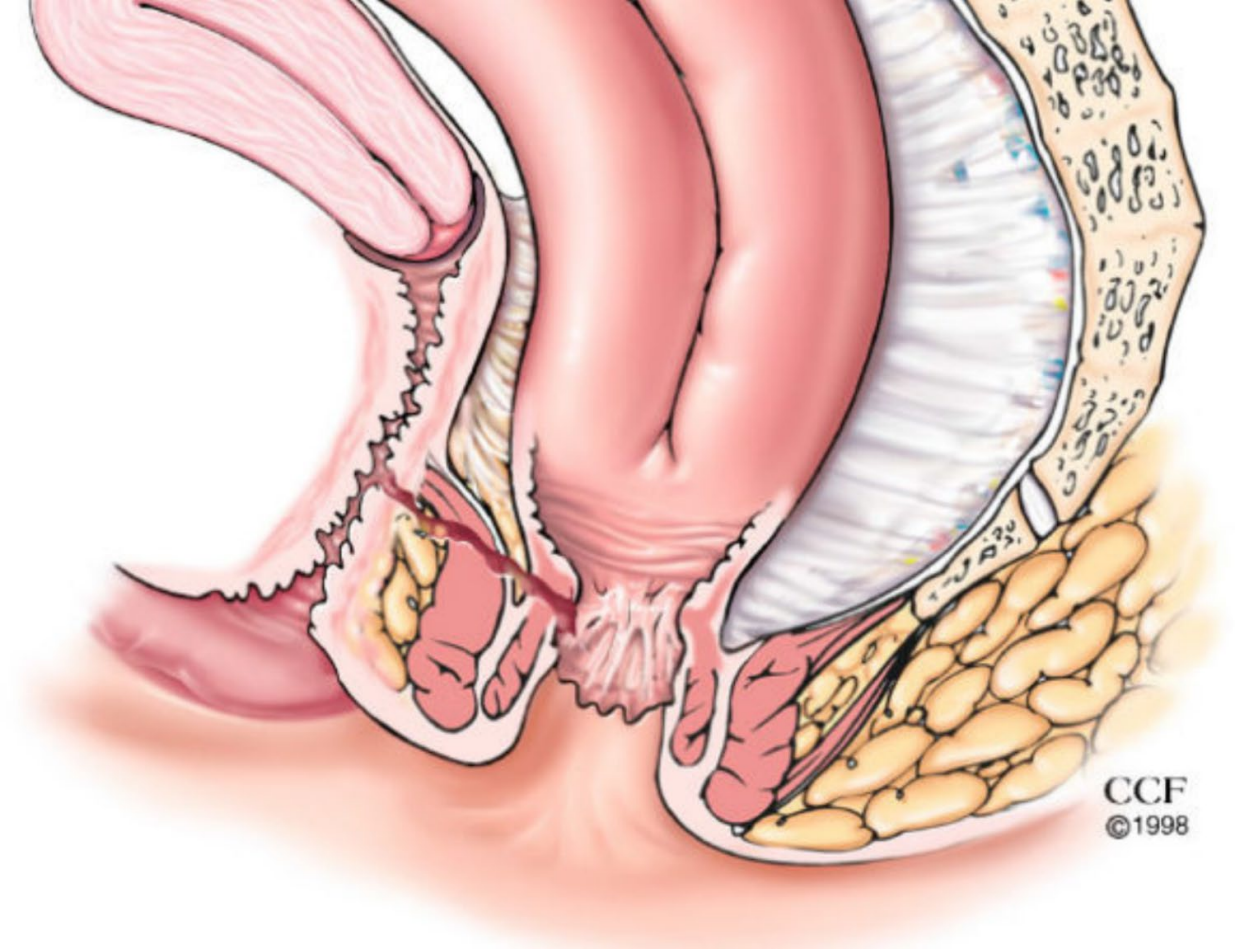
Illustration of pouch vaginal fistula

**Fig. 2:**
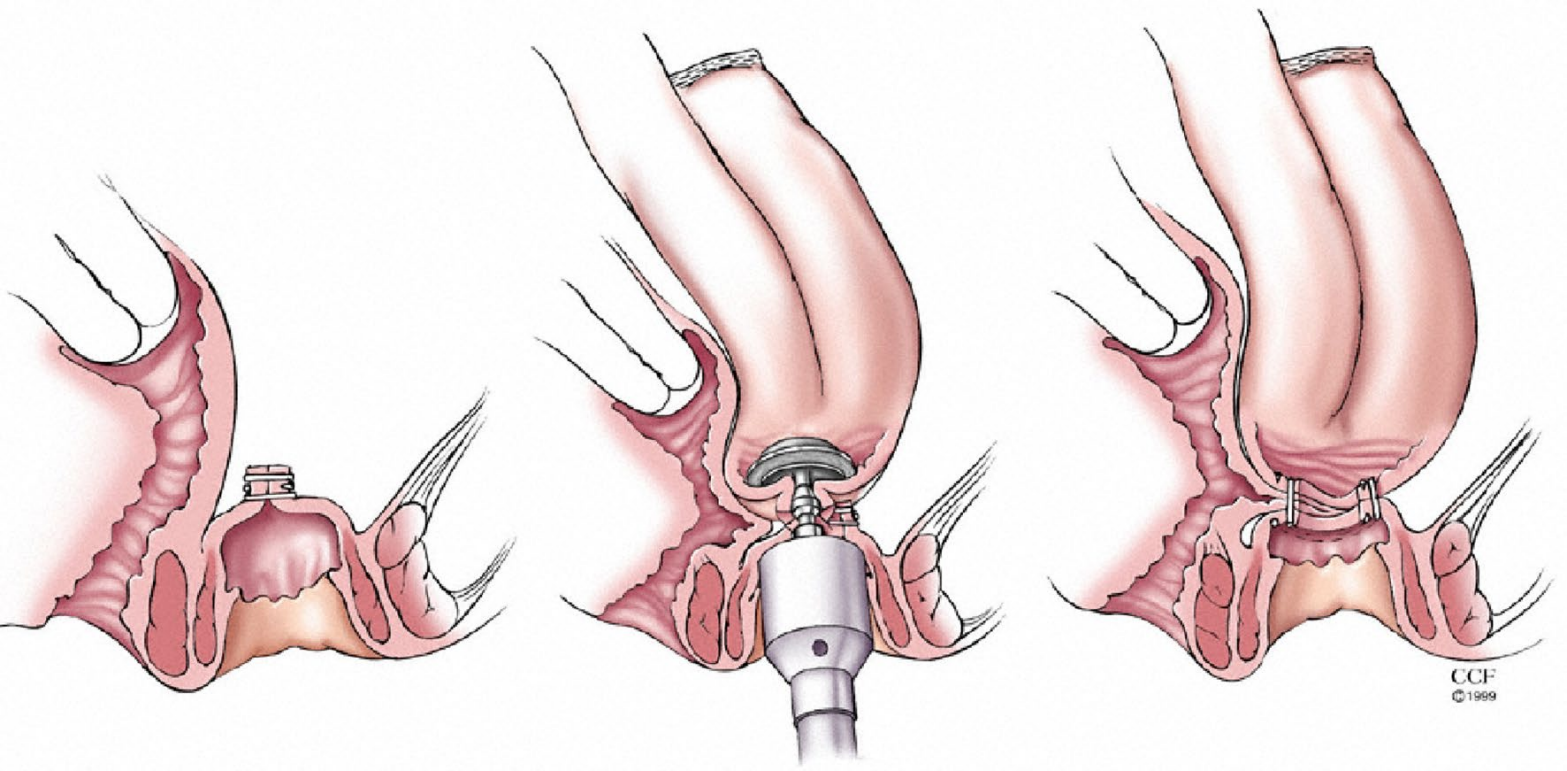
Inadvertent stapling of the back wall of the vagina into the pouch-anal anastomosis

Pouch vaginal fistulae (PVF) occur in approximately 6% of women after ileal pouch anal anastomosis (IPAA) and are challenging to manage (Fig. 1) [1–3]. The etiology of this complication is related to (a) technical aspects of surgery, e.g., inadvertently incorporating the posterior vaginal wall during stapling the IPAA (Fig. 2); (b) as sequelae of anastomotic leak, or (c) development of Crohn’s disease (CD) [3, 4]. Management ranges from conservative measures, e.g., stool bulking, seton placement, to transperineal or transvaginal local repairs, gracilis interposition flaps, and transabdominal approaches, e.g., redo pouch or pouch excision. Success rates are variable and typically less than 60% in patients with inflammatory bowel disease, with high recurrence rates [4, 5].

Circumferential pouch advancement (CPA) involves disconnecting the pouch from the anastomosis, transanally dissecting the distal pouch, advancing healthy pouch below the internal opening, and hand sewing the advanced pouch to the dentate line [6]. Although CPA has been previously described, limited data on the indications and clinical outcomes following this procedure is available [6–9]. We aimed to describe the indications and outcomes of CPA for PVF.

## Methods

Patients with IPAA who underwent CPA for any indication from 2009 to 2021 were identified from our pouch registry. Patients undergoing mucosal advancement flaps, or vaginal advancement flaps, were excluded. Demographics, operative variables, and outcomes were retrospectively reviewed. Early fistula was defined as occurring within 1 year after index IPAA, whereas late fistulae developed more than 1 year after IPAA. Clinical success was defined as resolution of symptoms, pouch retention, and no stoma at last follow-up. Figures represent frequencies (proportions) or medians (interquartile ranges).

### Technique

As reported by Fazio, anal eversion sutures are placed, and the anal canal mucosa proximal to the dentate line is typically lifted with submucosal infiltration of dilute epinephrine in saline. An anterior 180° or full 360° circumferential incision is used to perform a mucosectomy to the level of the anastomosis [6]. The pouch above the anastomosis is then dissected to expose the underlying sphincters, and careful circumferential dissection continued into the supralevator space to enable delivery of the pouch to the anal verge. The mucosectomy and peri-anastomotic tissue, including the internal opening, is trimmed off, and the internal vaginal opening closed using 2–0 absorbable sutures. The pouch is then advanced down to the dentate line, where it is handsewn in place using 2–0 absorbable sutures. Fecal diversion with temporary loop ileostomy, if not already present, is typically performed, and the patient receives a 2-week course of antibiotics. Patients typically undergo a contrast enema examination of the pouch and examination under anesthesia with pouchoscopy 3 months postoperatively, prior to ileostomy reversal.

## Results

### Baseline characteristics

Over 12 years, nine women underwent CPA for PVF; six were 360°, and three were 180° CPAs. The median age at IPAA and CPA was 28.0 (27–33) years and 41 (36–44) years, respectively, and the median body mass index (BMI) was 25.7 (22–26.8) kg/m^2^. After index IPAA, 4 (44.4%) patients developed early PVF, and 5 (55.6%) developed late PVF. The median time to fistula presentation was 32 (12–108) months. All patients had the diagnosis of ulcerative colitis at index IPAA; we did not observe any cases of familial adenomatous polyposis in this case series. All (5/5) late fistula patients were re-diagnosed to CD based on endoscopic appearance and biopsies. Fistulae were peri-anastomotic in 4 (44.4%) patients, one of whom also had an anovaginal fistula (AVF). The remaining known fistulas (4; 44.4%) were anovaginal in location. All patients had prior fistula procedures, including seton placement in 7 (77.8%), loop ileostomy (DLI) in 3 (33.3%), mucosal advancement flaps in 2 (22.2%), and fistula plug in 2 (22.2%). We did not observe any PVFs treated with over-the-scope clips. At time of CPA, 5 (55.6%) were on biologics.

### Operative interventions and outcomes

In terms of surgical intervention (Table 1), 4 (44.4%) patients had a DLI already present, 3 (33.3%) had a DLI constructed at the time of CPA, and 2 (22.2%) patients did not have a stoma at any point. Postoperative complications occurred in 3 (33.3%) patients, including high stoma output, urinary retention, and postoperative hematoma requiring operative intervention. The median follow-up time after CPA was 11 (6–24) months. Clinical success was achieved in 4/9 (44.4%) of patients at the time of last follow-up. Four of seven (57.1%) patients with DLI had reversal at last follow-up, three of whom had initial clinical success. The median interval from CPA to DLI reversal was 4.5 months. One of four (25%) patients who underwent reversal was promptly rediverted due to symptom recurrence. Both patients who did not have a DLI ultimately had their pouch excised or required temporary diversion.

**Table 1:** Descriptive details and outcomes of patients who underwent pouch advancement flap for pouch vaginal fistula. Four of the nine (44.4%) patients had clinical success (reversal of ostomy with no PVF symptoms) at a mean follow-up of 11 (6–24) months

#	Indication	Original/current diagnosis	PVF Onset	Location of PVF	Previous procedures	Cuffitis or anal stricture	Biologic at time of repair	Diverting loop ileostomy status	Complications	Follow-up, months	Time to DLI-R, months	Success?
1	PVF	UC/UC	Early	Distal to anastomosis	Anal mucosal advancement flap, vaginal advancement flap, DLI	Yes	Adalimumab	Present	None	24	NA	No*
2	PVF	UC/UC	Early	Distal to anastomosis	Anal mucosal advancement flap	Yes	No	Constructed	High stoma output	6	4	No**
3	PVF	UC/CD	Early	Unspecified	Seton placement	No	Infliximab-abda	No stoma	None	6	NA	No**
4	PVF	UC/CD	Early	Distal to anastomosis	Seton placement, fistula plug	Yes	Adalimumab	Present	None	3	NA	No***
5	PVF	UC/CD	Late	Anastomotic & distal to anastomosis	Seton placement	No	Golimumab	Constructed	None	24	5	Yes
6	PVF	UC/CD	Late	Anastomotic	DLI, seton placement	No	Certolizumab pegol	Present	None	11	6	Yes
7	PVF + stricture	UC/CD	Late	Anastomotic	Dilation, DLI	No	No	Present	Hematoma, urinary retention	4	3	Yes
8	PVF	UC/CD	Late	Anastomotic	Seton placement	No	No	No stoma	None	103	NA	No***
9	PVF	UC/CD	Late	Distal to anastomosis	Fistula plug	Yes	No	Constructed	Urinary retention	19	NA	Yes

*PVF* pouch vaginal fistula, *CD* Crohn’s disease, *UC* ulcerative colitis, *DLI* diverting loop ileostomy, *DLI-R* diverting loop ileostomy reversal

^*^Distal stoma limb stapled off

^**^Re-diverted due to symptomatic recurrence

^***^Pouch excised, and end ostomy created

## Discussion

Pouch vaginal fistulae, which are highly symptomatic and distressing for patients, are difficult to treat and often an indication for pouch revision or excision. We found that CPA was a viable option for patients who had failed previous interventions for PVF repair, with 44% of patients living stoma-free and fistula-free at the time of follow up. In a meta-analysis of PVF by Machin et al., a total of 577 PVFs from 13 studies were reviewed, with an PVF incidence rate of 2.1–17.1% [1]. They observed an overall success rate for abdominal vs. local repairs of 60.2% and 44.9%, respectively.

Despite the limited success rate of transanal repairs compared with transabdominal repairs observed in this meta-analysis, transanal sphincter-preserving options are attractive to surgeons and patients alike to avoid the increased morbidity invariably associated with transabdominal repair. As with any handsewn anastomosis, patients undergoing CPA should be counseled regarding the possibility of nocturnal seepage, particularly when deeply asleep. The success rate of CPA is likely improved with the protection of a diverting loop ileostomy, as prior studies have demonstrated its effectiveness in aiding the healing process, especially if initial treatment attempts have failed. While stoma utilization was very high in our study (77.8%), our data are limited in number to definitively state that the CPA success rate is significantly impacted by the presence of a defunctioning ostomy. The two un-diverted patients in our study eventually required temporary DLI or pouch excision, suggesting that diversion at the time of CPA may have been beneficial. Both diversion and seton placement prior to CPA were left to surgeon discretion. Notably, 55% of our patients had draining setons in place at the time of CPA, but it is unclear if this is required prior to CPA; of those with setons prior to CPA, the healing rate was 40%.

To date, few studies have been published on full thickness transanal CPA for PVF, other fistulae, or anal canal strictures. Fazio and Tjandra first described transanal mucosectomy with pouch advancement for dysplasia or cuffitis in 1993 in two patients [6]. In terms of outcomes, both patients had intact continence with nocturnal minor seepage of liquid stool, and neither developed a stricture. In 2001, Zmora et al. described eight patients who underwent CPA, four of whom required transabdominal mobilization as well, with a success rate of 62% which is slightly higher than our rate of 44.4% [7]. Similar to Fazio et al*.*, they reported good functional results in 63%. Mallick et al. also described a 50% success rate with utilization of pouch advancement flaps [9]. Anal canal strictures may also be treated with CPA, as reported by Prudhomme et al. in 2003, who reported on five patients who required transanal excision of the stricture and mucosal advancement in which the strictured segment was excised and a flap of ileal mucosa advanced [8]. CPA may not only have a place in the management of PVF but may also serve as a viable option for additional perianal pathologies, especially recalcitrant strictures in this high-risk population of patients with inflammatory bowel disease.

The etiology and timing of the development of a PVF may impact the success of CPA. PVF may occur because of inadvertent incorporation of the posterior vagina wall into the pouch-anal anastomosis during firing of the circular stapler (Fig. 2) [1, 2]. An early PVF at the level of the anastomosis would be the expected result of this technical complication, although paradoxically all the anastomotic PVF in our study presented late suggesting this was not the case. All these patients were ultimately diagnosed with CD, which may account for this delayed presentation. The late development of these fistulae (32 months) suggests CD was the etiology for the majority. More distal manifestations of vaginal fistulas may be a result of inflammation and fistulization related to cuffitis or CD of the anal canal. If present, such inflammation mandates systemic treatment with biologic or small-molecule medications prior to the attempted repair. While patients with anastomotic PVF and those with fistulae distal to the anastomosis both had clinical success in our study, it remains unclear if CPA success is dependent on the location of the fistula [3].

Our study has several limitations. As a result of its retrospective nature, the technical aspects of the CPA were derived from operative reports which may have been limited in detail. The long study period and number of operating surgeons may have resulted in variation in techniques and outcomes. Technical issues, such as inadvertent stapling of the vagina during IPAA creation, were unable to be determined retrospectively. Patients’ follow-up was also limited to our own medical records, and functional outcomes and long-term follow-up (> 1 year) were limited.

## Conclusion

Full thickness transanal circumferential pouch advancement is a viable treatment for refractory pouch vaginal fistulae and may be an option for patients who have undergone multiple attempts at fistula repair.

## Data Availability

The data generated during this study are available from the corresponding author upon reasonable request. No datasets were generated or analysed during the current study.

## Abbreviations

AVF: Anovaginal fistula
BMI: Body mass index
CD: Crohn’s disease
CPA: Circumferential pouch advancement
DLI: Diverting loop ileostomy
IPAA: Ileal pouch anal anastomosis
PVF: Pouch vaginal fistulae
